# Testing the arousal hypothesis of neonatal imitation in infant rhesus macaques

**DOI:** 10.1371/journal.pone.0178864

**Published:** 2017-06-15

**Authors:** Annika Paukner, Eric J. Pedersen, Elizabeth A. Simpson

**Affiliations:** 1 *Eunice Kennedy Shriver* National Institute of Child Health and Human Development, National Institutes of Health, Poolesville, Maryland, United States of America; 2 Department of Psychology, University of Miami, Coral Gables, Florida, United States of America; 3 Department of Psychology and Neuroscience, University of Colorado Boulder, Boulder, Colorado, United States of America; University of Portsmouth, UNITED KINGDOM

## Abstract

Neonatal imitation is the matching of (often facial) gestures by newborn infants. Some studies suggest that performance of facial gestures is due to general arousal, which may produce false positives on neonatal imitation assessments. Here we examine whether arousal is linked to facial gesturing in newborn infant rhesus macaques (*Macaca mulatta*). We tested 163 infants in a neonatal imitation paradigm in their first postnatal week and analyzed their lipsmacking gestures (a rapid opening and closing of the mouth), tongue protrusion gestures, and yawn responses (a measure of arousal). Arousal increased during dynamic stimulus presentation compared to the static baseline across all conditions, and arousal was higher in the facial gestures conditions than the nonsocial control condition. However, even after controlling for arousal, we found a condition-specific increase in facial gestures in infants who matched lipsmacking and tongue protrusion gestures. Thus, we found no support for the arousal hypothesis. Consistent with reports in human newborns, imitators’ propensity to match facial gestures is based on abilities that go beyond mere arousal. We discuss optimal testing conditions to minimize potentially confounding effects of arousal on measurements of neonatal imitation.

## Introduction

Human newborns are responsive to their environments by modifying the movements of their mouths: they alter their suckling patterns for sweet solutions [1], and they respond to the odor of their mother’s breast milk by making mouthing movements [2]. Newborns also use their mouths to interact with visual stimuli: Meltzoff and Moore [3–4] found that within minutes after birth, infants respond to facial gestures with matching facial gestures. The most commonly tested facial gestures are mouth opening and tongue protrusion, but newborns also match other visual stimuli, e.g. lip protrusion [5], emotional facial expressions [6], head movement [7], and even finger movements [8–9]. Overall, results have been mixed with some researchers reporting positive evidence of matching behaviors, and others failing to find evidence of what has been termed neonatal imitation. There are multiple reasons why some studies may have failed to replicate previous findings, ranging from testing infants outside of windows of a sensitive period [10] to methodological shortcomings (for a discussion, see [11]). A meta-analysis by Anisfeld [12–13] found that the most consistently–and some may argue the only–matched gesture is tongue protrusion, which raises the question of whether tongue protrusion matching is a case of imitation or whether alternative accounts may be sufficient to explain this phenomenon.

Imitation has been defined in numerous ways, emphasizing the matching of either the form of movements, the outcomes, or the intentions of an act (e.g. see [14]). For the current purposes, we will focus on a newborn’s ability to match the behaviors of others in motoric form following the observation of the same behavior by a model, without making any assumptions regarding the infant’s motivation or intention. Behavior matching can be achieved via visual-visual matching, or in the case of facial gestures, visual-motor matching. That is, being unable to see their own faces, newborns must match the visual input of someone else’s facial gesture with the kinesthetic and proprioceptive feedback of their own faces. Since this achievement spans at least two sensory modalities, Meltzoff and Moore [15] proposed that infants are born with a supramodel representation of acts, which facilitates this visuo-motor matching of behaviors. If newborns are able to match numerous gestures, then the idea of a general ‘active intermodal mapping’ (AIM; [15] appears plausible. If, on the other hand, newborns increase the frequency of one particular gesture (such as tongue protrusion) in response to various visual stimuli, it would be more parsimonious to conclude that tongue protrusion is part of the newborn’s general arousal response, which by coincidence happens to resemble a matching response when performed after seeing a tongue protrusion model [3]. It is therefore important to compare the responses in the matching condition with the responses in a control condition, and imitation should only be inferred when the performance in the matching condition is greater than performance in the non-matching condition [3]; otherwise, arousal cannot be ruled out as an alternative explanation.

The idea that tongue protrusion is merely one of neonates’ exploratory or arousal responses has been explored by Jones [16–17]. Jones presented newborns with blinking lights [16] and music [17] and observed that tongue protrusions increased in both test situations. Nagy and colleagues [18–19] tested in more detail whether tongue protrusion gestures may be linked to infant arousal. Nagy and Molnar [18] found that newborn heart rates accelerate when infants perform tongue protrusion gestures while viewing a tongue protrusion model, suggesting that the act of protruding their tongues may indeed involve an element of arousal for newborns. However, Nagy et al. [19] also measured other potential indicators of arousal such as arm and finger movements and general state of alertness; none of these were found to increase over the course of the test session, or when presented with a tongue protrusion model compared to a baseline measurement. Instead, they report that newborns in the first 5 days of life increased their *strong* tongue protrusions (i.e., fully extended tongues beyond the lips), but not their *weak* tongue protrusions (i.e., tongue visible but not protruding beyond the lips), while observing a tongue protrusion gesture compared to a baseline period; the latter type of gesture may be more of an arousal response, but the former appears selectively imitative [19]. Thus, while the evidence to date indicates that arousal may be associated with a tongue protrusion response, it still remains unclear whether arousal on its own is sufficient to explain facial gesture matching in newborns.

Interestingly, other species also show neonatal imitation responses. Bard [20] reports imitation of mouth opening gestures (but not tongue protrusion gestures) in infant chimpanzees in both a structured and a communicative testing paradigm, and suggests that this propensity may be linked to the species-typical smile or play face. Similarly, infant rhesus macaques imitate lipsmacking gestures (rapid opening and closing of the mouth) [21], which is a communicative gesture conveying affiliation between mothers and infants. Macaque infants who match lipsmacking gestures also perform more lipsmacking gestures after a delay [22], specifically when the lipsmacking model is the same person rather than a new person [23]. Is it possible that in nonhuman populations, matching of facial gestures are an artefact of arousal and that the match with the model’s gestures is merely coincidental?

In the present study, we aimed to determine whether facial gestures may be the result of arousal in infant rhesus macaques, and consequently whether arousal could be the responsible mechanism of so-called imitative behavior. To measure arousal, we counted yawning during the neonatal imitation task. Although finger and arm movements may be a good indicator of arousal in human infant studies [19], and scratching has associated with arousal in macaques [24], infant macaques are frequently swaddled in soft blankets or cloths during testing, thus finger and arm movements may be inhibited or, at least, difficult to observe. Yawning, on the other hand, is generally associated with arousal in a variety of animals. For example, when administered experimentally, adrenocorticotropic hormone (ACTH), a pituitary hormone that stimulates the release of cortisol, a stress hormone, facilitates yawning in dogs, cats, rabbits, and mice [25]. In non-human primates, several studies have suggested that yawning increases in situations of stress or increased arousal [26–31]. Developmentally, Nazka booby (*Sula granti*) nestlings show increased yawning rates following natural maltreatment by non-parental adults [32] and in humans, pre-term infants’ yawning has been associated with increased behavioral arousal [33] as well as painful procedures such as heel lance for blood collection [34], suggesting that yawning and arousal are associated from early on in life and making it a suitable measure for the current study.

Below we describe infant macaques’ yawning responses and their association with facial gestures (lipsmacking and tongue protrusion) in a neonatal imitation task. Infants were presented with three different stimuli, a lipsmacking condition (LPS, an affiliative facial gesture), a tongue protrusion condition (TP, no communicative meaning), and a nonsocial control condition (CTRL, a white plastic disk with high contrast orthogonal stripes). To determine whether facial gestures may be the result of arousal, we first examined arousal (measured by yawning frequencies) across conditions to determine whether the conditions containing facial stimuli (LPS and TP) may be more arousing than the CTRL condition. Moreover, past studies have inferred imitation in matching facial gestures if there is an increase between a (static/still) Baseline and a (dynamic) Stimulus phase, which is larger in the facial gesture condition than the spinning-disk control condition [20]; we therefore examined whether arousal (yawning) would show a similar increase between Baseline and Stimulus phases specifically in the LPS and TP conditions but not the CTRL condition. We then identified subsets of infants who showed an increase in lipsmacking responses between the Baseline and the Stimulus phase specifically in the LPS condition, called LPS Imitators, or an increase in tongue protrusion gestures between Baseline and Stimulus specifically in the TP condition, called TP Imitators. We compared these infants’ levels of arousal across conditions and to infants who did not show an increase facial gestures (Non-imitators), hypothesizing that if facial gestures are caused by arousal, Imitators should show higher levels of arousal than Non-imitators, specifically in the facial gesture conditions. Finally, given the assumed association between arousal and facial gestures, we investigated whether changes in yawning between Baseline and Stimulus could explain the increases in the selective matching of lipsmacking and tongue protrusion responses of Imitators in the facial gesture conditions.

## Materials and methods

### Ethics statement

This study was reviewed and approved by the Eunice Kennedy Shriver National Institute of Child Health and Human Development Animal Care and Use Committee. The study was conducted in accordance with the Guide for the Care and Use of Laboratory Animals and complied with the Animal Welfare Act. During the course of this study, infants were exclusively fed with Similac^®^ Advance^®^ (Abbott Laboratories). Infants’ housing was enriched by an inanimate surrogate mother covered with fleece fabric as well as blankets and various plastic and rubber toys, which were rotated daily. At the conclusion of data collection for the current study, infants continued to be housed in the nursery as part of ongoing, unrelated research studies until ca. 6–8 months of age, after which they were transferred to large peer groups. See [35] for further details on housing, enrichment, and feeding.

### Subjects

Subjects were 163 infant rhesus macaques (*Macaca mulatta*, 88 males). All infants were separated from their mothers on the day they were born (typically by 8am), and were reared in a nursery facility for ongoing, unrelated research studies. Infants were individually housed in incubators (51 cm × 38 cm × 43 cm) for the first two weeks of life and in metal cages thereafter. For the first month of life, infants could see and hear, but not physically contact, other infants of similar age. Human caretakers were present for 13h each day and interacted with infants every 2h for feeding and cleaning purposes. Imitation data from 126 infants were previously published as part of other studies [22, 35–41]. We used all available data and did not exclude infants based on imitation or yawning performance.

### Procedure

Infants were tested for neonatal imitation three times a day, every other day, in the first week of life (days 1–2, 3–4, 5–6, and 7–8), for up to four days. In total, 134 infants were tested on 4 days, 25 infants were tested on 3 days, and 4 infants were tested on 2 days. There was at least an hour between each test session. Infants were tested within an hour of feeding to ensure they were not fussy due to hunger. Infants had to have their eyes open and had to show signs of active orientation towards stimuli appearing in their visual field (e.g., the experimenter approaching the incubator). Infants were transported from their incubator to a nearby testing room. During transportation to the testing room, infants clung to the arm of the experimenter, and had to show signs of body reactivity towards changes in posture and support instability. Failure to show these signs of alertness resulted in a delay of the test session until, at a later time in the same day, infants were sufficiently alert. One experimenter held each infant on her lap, often swaddled or loosely wrapped in a diaper pad or soft fleece. All infants were awake and alert (but not distressed) at the beginning of the test. A demonstrator presented infants with three stimuli, one during each session, at a distance of approximately 30 cm at eye-level with the infant: a lipsmacking gesture (LPS, rapid opening and closing of the mouth), a tongue protrusion gesture (TP, slow protrusion and retraction of the tongue, ca. 1 gesture per second), and a nonsocial control condition (CTRL, a white plastic disk with orthogonal stripes—which were either black/red or green/yellow—slowly rotated clockwise and counter-clockwise). Each stimulus type was presented once a day to infants; the order of stimulus presentations remained the same for each infant but was randomized between infants. In each test session, one experimenter held the infant, a second experimenter—the demonstrator—served as the source of the stimuli, and a third experimenter was the time-keeper who ensured stimuli were presented for appropriate lengths. All sessions were videotaped. Individual demonstrators were randomly assigned to conditions but remained consistent across days within each infant. Twenty seven infants were only tested in the LPS and CTRL conditions, not the TP condition, and 4 sessions were lost due to experimenter error, leaving a total of 1,745 sessions.

At the beginning of a trial, a 40 sec baseline was conducted, in which the demonstrator displayed a calm, neutral facial expression (or the still disk in control). During the subsequent stimulus period, the demonstrator displayed a facial movement (lipsmacking or tongue protrusion) or rotated the disk for 20 seconds, followed by a still/neutral facial expression (still disk in CTRL) period for 20 seconds. This movement-still face sequence was repeated and ended with an additional 20 second movement phase (total of 100 second stimulus period). See supplemental materials for video examples.

### Analyses

Infants’ mouth movements were coded off-line, frame-by-frame (≥30 frames per second), for lipsmacking gestures, tongue protrusion gestures, and yawns. Lipsmacking was operationally defined as a high frequency opening and closing of the mouth without sound production in which the lips were required to part and then rejoin within 2 seconds. Tongue protrusions were defined as forward movements of the tongue so that it crossed the inner edge of the lower lip. Yawns were coded when infants performed a full mouth stretch which could (but did not necessarily have to) last for more than 2 seconds, sometimes combined with closing of the eyes. Observers were blind to the stimulus. Inter-observer reliability was assessed between an anchor observer and one additional observer for 345 sessions (19.8% of total sessions). Inter-observer agreements were high for all facial gestures (lipsmacking: *M*_*1*_ = 9.97, *M*_*2*_ = 10.61, intraclass correlation coefficient (ICC) = .92, 95% CI (.90, .93); tongue protrusion: *M*_*1*_ = 9.71, *M*_*2*_ = 10.24, ICC = .93, 95% CI (.92, .95); yawns: *M*_*1*_ = 1.18, *M*_2_ = 1.12, ICC = .97, 95% CI (.96, .98).

Unless otherwise stated, the main analyses were conducted using Bayesian generalized linear mixed models (GLMMs) with overdispersed Poisson distributions to properly model yawns as counts [42–43]. The reason for using these analyses was the severely positive skewed distribution of yawns when accounting for individual test days, which would not be appropriately handled by analyses of variance (ANOVA). We ran models in R version 3.2.3 [44] via the MCMCglmm package [45]. To account for repeated measures, these models have random intercepts for subjects and random slopes for test day and condition, and all were run with non-informative priors. The coefficients for the models were log rates and were interpreted as rate ratios (RRs) when exponentiated. We report 95% highest density intervals (HDIs) for the coefficient estimates and pMCMC (a Bayesian “p-value” based on Markov chain Monte Carlo estimation, defined as two multiplied by the probability that the parameter value is less than or greater than zero, using the smaller of these probabilities; [45]). We consider a result statistically significant if (a) the 95% HDI did not include zero and (b) pMCMC was less than .05.

## Results

### Sex differences in yawning

Since yawning rates are thought to be sexually dimorphic in adult rhesus macaques [46], we first checked whether we would have to control for this variable by examining whether male and female infant rhesus macaques differed in their yawn frequencies. A GLMM predicting total yawns per test day with dummy-coded predictors for sex, condition, and their interactions found no effect for sex and no interactions (all pMCMCs > 0.139). Sex was therefore not included in subsequent analyses.

### Yawning across test days

Neonatal imitation tests were performed over the course of the infants’ first week of life, leaving open the possibility that yawning rates may change during this period of time. While not the main variable of interest in the current study, any change over time could nonetheless potentially bias the analyses. We therefore examined yawns per session across test days using a GLMM predicting total yawns per session with predictors for condition, day, and their interaction. Day was defined as a continuous variable with the intercept set to be the first test day (day 1 or 2 of life). There was a significant amount of yawning on day 1 in the CTRL condition (i.e., the intercept), b = -1.55, 95% HDI [-1.86, -1.23], pMCMC < .001. Exponentiating the intercept (e^-1.55^) returned a rate ratio (RR) of .21, indicating that the model-predicted number of yawns per session on day 1 was .21. There was also a significant effect for day, b = .16 (RR = 1.17), 95% HDI [.09, .22], pMCMC < .001, indicating that each additional day lead to a 17% increase in predicted yawns per session. Day did not interact with condition, indicating that the effect of day was consistent across conditions. Thus, yawn rates significantly increased with test day over the first week of life (means: day 1/2: 0.48, day 3/4: 0.75, day 5/6: 1.04, day 7/8: 1.16). To account for this change over time in the model, in all subsequent analyses, unless otherwise indicated, we included day as a random effect, but we did not include it as a fixed effect or test for interactions with other effects.

### Occurrences of yawns in relation to trial phases and condition

Each neonatal imitation trial was divided into a static Baseline followed by a dynamic-static (burst-pause) Stimulus phase. Since the arousal hypothesis purports that facial gestures are the result of arousal, we first examined levels of arousal (as measured by yawn frequencies) during the Baseline and Stimulus phases to determine whether a particular condition may be more arousing than the others.

#### Baseline phase

**Fig 1:**
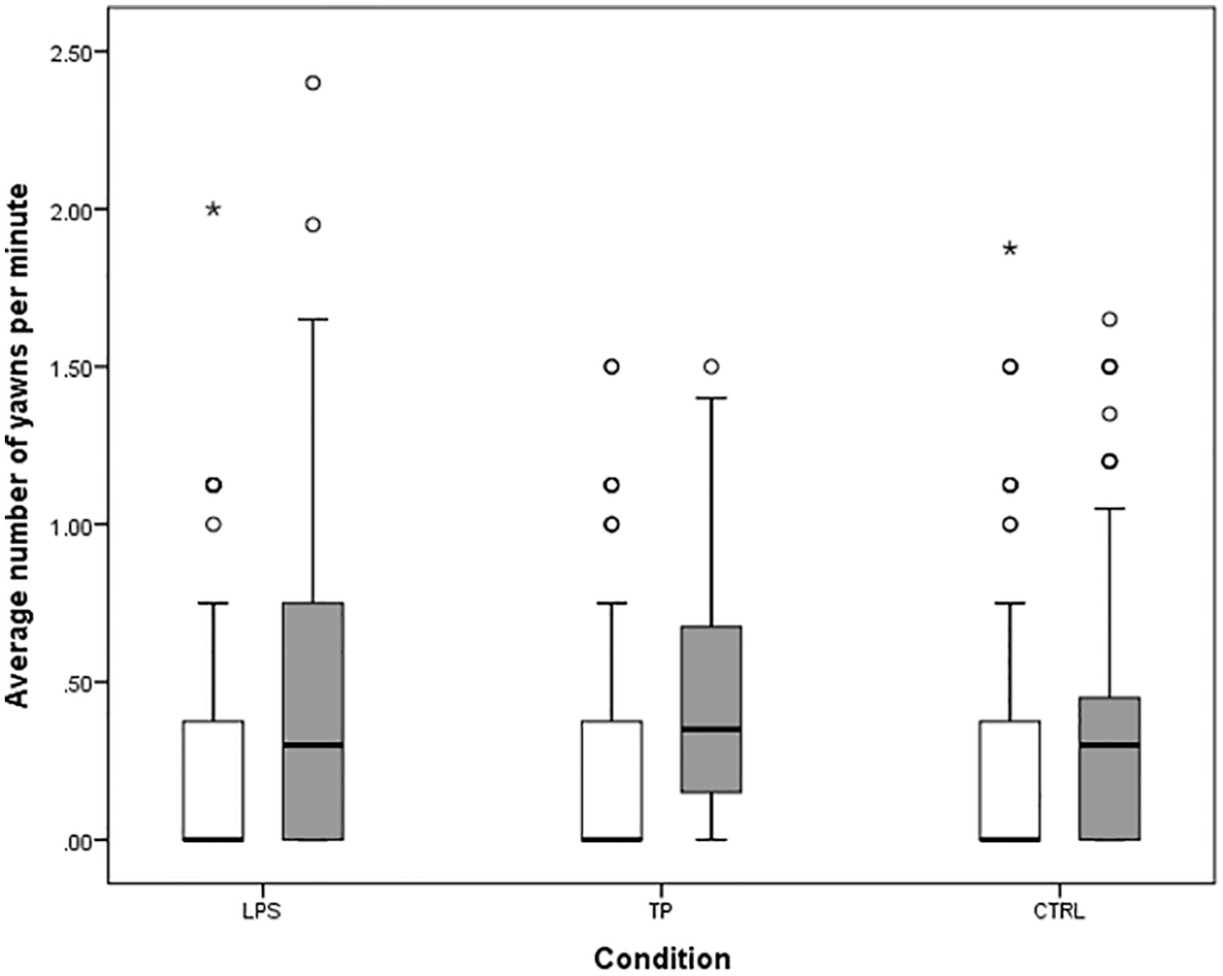
Boxplots of the average rate of yawning per minute during Baseline (white) and Stimulus (grey). LPS = lipsmacking condition, TP = tongue protrusion condition, CTRL = control condition. Boxplots depict 1^st^ and 3^rd^ quartile, line depicts median, whiskers depict 1.5 times inter-quartile range (IQR). Circles represent mild outliers larger than Q3 + 1.5 * IQR, asterisks represent extreme outliers larger than Q3 + 3 * IQR.

We explored whether there were differences across conditions during the Baseline period with a Bayesian linear mixed model predicting yawns during Baseline (averaged across test day; low variance in the number of yawns per baseline period per day led to unstable parameter estimates in a count model similar to our other analyses, though the results were directionally similar to what we report here), and included random intercepts for subjects and random slopes for condition. Because the stimulus during the Baseline phase was identical for the LPS and TP conditions (a still face), we jointly compared these two face conditions against the CTRL condition with a dummy-coded predictor. There was a small but significant amount of yawning in the CTRL condition during Baseline, b = .14, 95% HDI [.10, .18], pMCMC < .001. There was not significantly more yawning in the face conditions, b = .03, 95% HDI [-.013, .067], pMCMC = .195, compared to the CTRL condition (Fig 1). Thus, the data suggest that static faces were not more arousing than a nonsocial control object.

#### Stimulus phase

We next explored the Stimulus phase, in which infants viewed dynamic stimuli (facial gestures or rotating disk), with a GLMM predicting the number of yawns during the Stimulus phase. Unlike in the Baseline phase, the stimuli were different in each condition, so we used separate dummy codes for the LPS and TP conditions. There was a significant amount of yawning during the Stimulus phase in the CTRL condition, b = -1.12 (RR = .33), 95% HDI [-1.32, -.93], pMCMC < .001. There were significantly more yawns in the LPS condition compared to CTRL, b = .23 (RR = 1.26), 95% HDI [.07, .39], pMCMC = .005, and there were significantly more yawns in the TP condition compared to CTRL, b = .23 (RR = 1.26), 95% HDI [.06, .40], pMCMC = .009. Re-running the model with dummy variables recoded to set LPS as the intercept showed that there was no significant difference between LPS and TP, b = .01, 95% HDI [-.17, .16], pMCMC = .998. Overall, during the Stimulus phase infants yawned approximately 26% more during LPS and TP than CTRL with no difference between LPS and TP (Fig 1), suggesting that dynamic facial displays may be more arousing than non-social dynamic displays, regardless of the specific facial gesture.

#### Change from Baseline phase to Stimulus phase across conditions

The previous analysis suggests that dynamic facial stimuli may be more arousing than the dynamic control object, which potentially holds implications for neonatal imitation of facial gestures. However, neonatal imitation is commonly assessed by evaluating the *increase* in matching responses between a Baseline and a Stimulus phase. If an increase in arousal were responsible for an increase in facial gestures, infants should also show a relatively larger increase in yawning between Baseline and Stimulus in the LPS and TP conditions compared to the CTRL condition. To test this hypothesis, we ran a Bayesian linear mixed model with dummy codes for condition (LPS and TP were coded separately), predicting a difference in the rate of yawns per minute during Stimulus versus Baseline (averaged across test day), and included random intercepts for subjects. There was a significant increase in the rate of yawns/min in the CTRL condition, b = .16, 95% HDI [.10, .22], pMCMC < .001. Neither the dummy code for LPS (b = .06, 95% HDI [-.01, .13], pMCMC = .115) nor the dummy code for TP (b = .03, 95% HDI [-.04, .11], pMCMC = .420) were significant, indicating that the increases in yawning between Baseline and Stimulus in the LPS and TP conditions were not different from the increase in yawning between Baseline and Stimulus in the CTRL condition. These results suggest that even though infants yawned more overall during the Stimulus phase of LPS and TP compared to CTRL, the change in yawning between Baseline and Stimulus did not differ across conditions. Given that imitation is inferred based on the difference in facial gestures between Baseline and Stimulus, this result does not support the idea that arousal increases specifically in the LPS or TP conditions and could thereby lead to increased facial gestures.

### Is arousal sufficient to explain facial gestures in all infants? Imitators vs. Non-imitators

The results above suggest that arousal may increase over the course of a neonatal imitation test as evidenced by the higher rate of yawning during the Stimulus phase, particularly in the LPS and TP conditions. However, the increases in yawning between Baseline and Stimulus were similar among all three conditions. Since imitation is inferred if a facial gesture increases between a Baseline and a Stimulus phase specifically in the condition where the facial gesture is presented to infants, the arousal hypothesis would predict an increase in arousal (i.e., yawning) also specifically in the matching condition, which we did not observe. Thus, while the results suggest that arousal may contribute to facial gestures being performed in the Stimulus phase, arousal is insufficient to explain why some infants increase facial gestures specifically in the matching condition. These infants, which we have called Imitators in the past, are identified by averaging lipsmacking or tongue protrusion gesture rates (per 60 sec) from Baseline and Stimulus across all test days in each condition. Infants are Imitators if they produce an increase in lipsmacking or tongue protrusion from the Baseline to the Stimulus phase in the LPS or TP condition to a greater extent than they increase lipsmacking or tongue protrusion from the Baseline to the Stimulus phase in the CTRL condition (see also [22; 23]). If arousal was responsible for this increase in facial gestures, then we would predict a higher frequency of yawning in Imitators compared to Non-imitators.

To explore this idea further, we assessed the imitator status of the current sample. We identified 79 lipsmacking Imitators and 84 lipsmacking Non-imitators, and 49 tongue protrusion Imitators and 87 tongue protrusion Non-imitators. We then repeated our above analyses to include Imitator / Non-imitator as a between-subject factor to explore whether Imitators and Non-Imitators differed in their general arousal level (as seen by a difference in yawning rates).

#### Baseline phase—Lipsmacking Imitators

**Fig 2:**
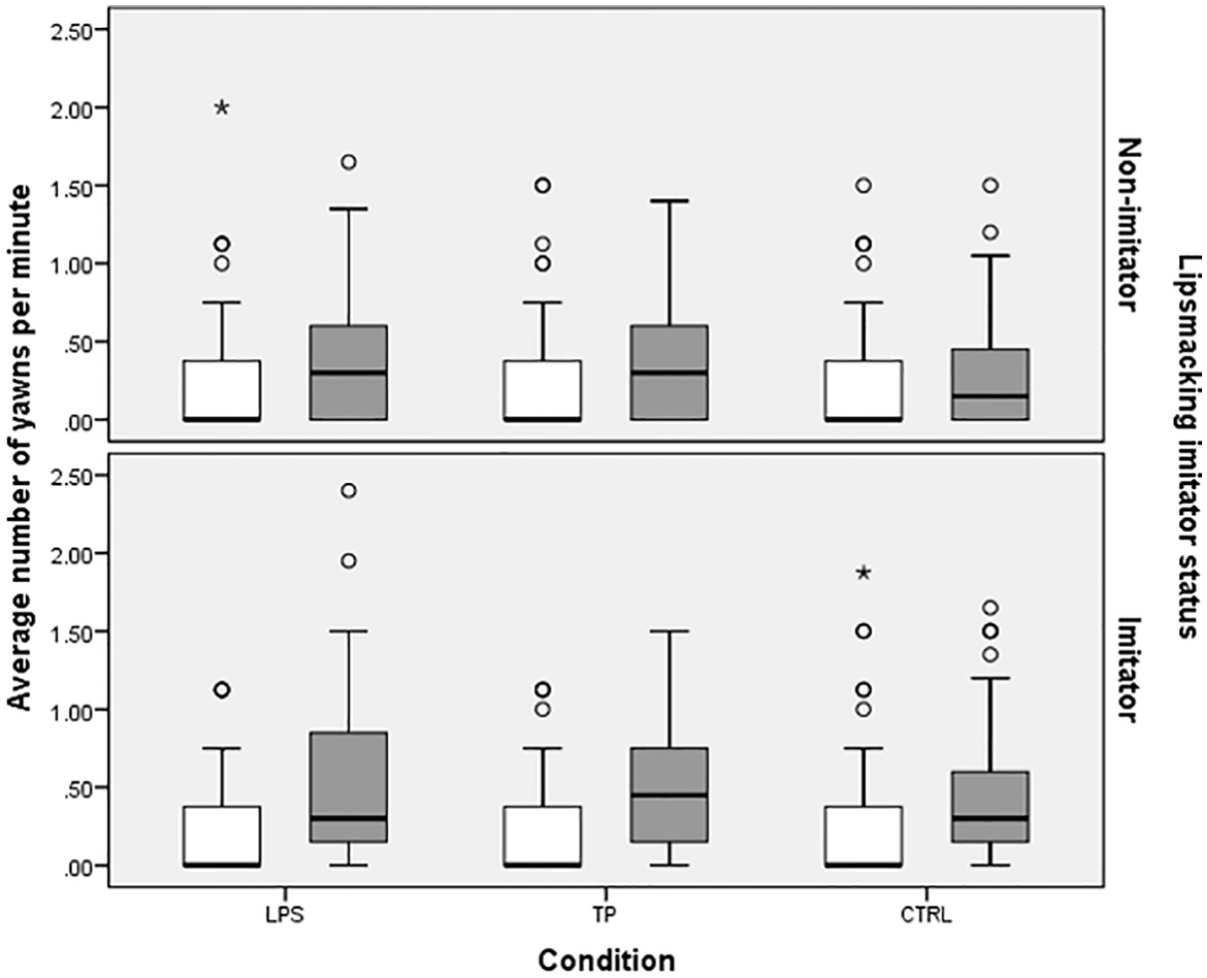
Boxplots of the average rate of yawning per minute during Baseline (white) and Stimulus (grey) for lipsmacking Non-imitators (top) and Imitators (bottom). LPS = lipsmacking condition, TP = tongue protrusion condition, CTRL = control condition. Boxplots depict 1^st^ and 3^rd^ quartile, line depicts median, whiskers depict 1.5 times inter-quartile range (IQR). Circles represent mild outliers larger than Q3 + 1.5 * IQR, asterisks represent extreme outliers larger than Q3 + 3 * IQR.

Pooling across all conditions, we explored whether Imitators and Non-imitators differed in their yawn rates during the Baseline period using a Bayesian linear mixed model with a dummy code for lipsmacking imitator status predicting yawns during Baseline (averaged across days; see footnote 1) with random intercepts for subjects and random slopes for condition. The intercept of the model was significant, b = .14, 95% HDI [.10, .19], pMCMC < .001, indicating that Non-imitators yawned a significant amount. There were no differences between Imitators and Non-imitators, b = .02, 95% HDI [-.03, .08], pMCMC = .418. Next, we added a dummy code for the face conditions (LPS and TP combined, since they were indistinguishable, i.e. both still faces during the baseline) versus the CTRL condition into the model, as well as a condition by imitator status interaction. The intercept of the model was significant, b = .11, 95% HDI [.06, .17], pMCMC < .001, indicating that Non-imitators in the CTRL condition yawned a significant amount. However, Non-imitators did not yawn significantly more in the LPS and TP conditions than in the CTRL condition, b = .05, 95% HDI [-.005, .11], pMCMC = .064. The dummy coded predictor for imitator status was not statistically significant, b = .06, 95% HDI [-.02, .14], pMCMC = .14, nor was the imitator status by condition interaction, b = -.05, 95% HDI [-.13, .03], pMCMC = .190, indicating that Imitators did not yawn more than Non-imitators in either the face conditions or the CTRL condition (Fig 2).

#### Baseline phase—Tongue protrusion Imitators

**Fig 3:**
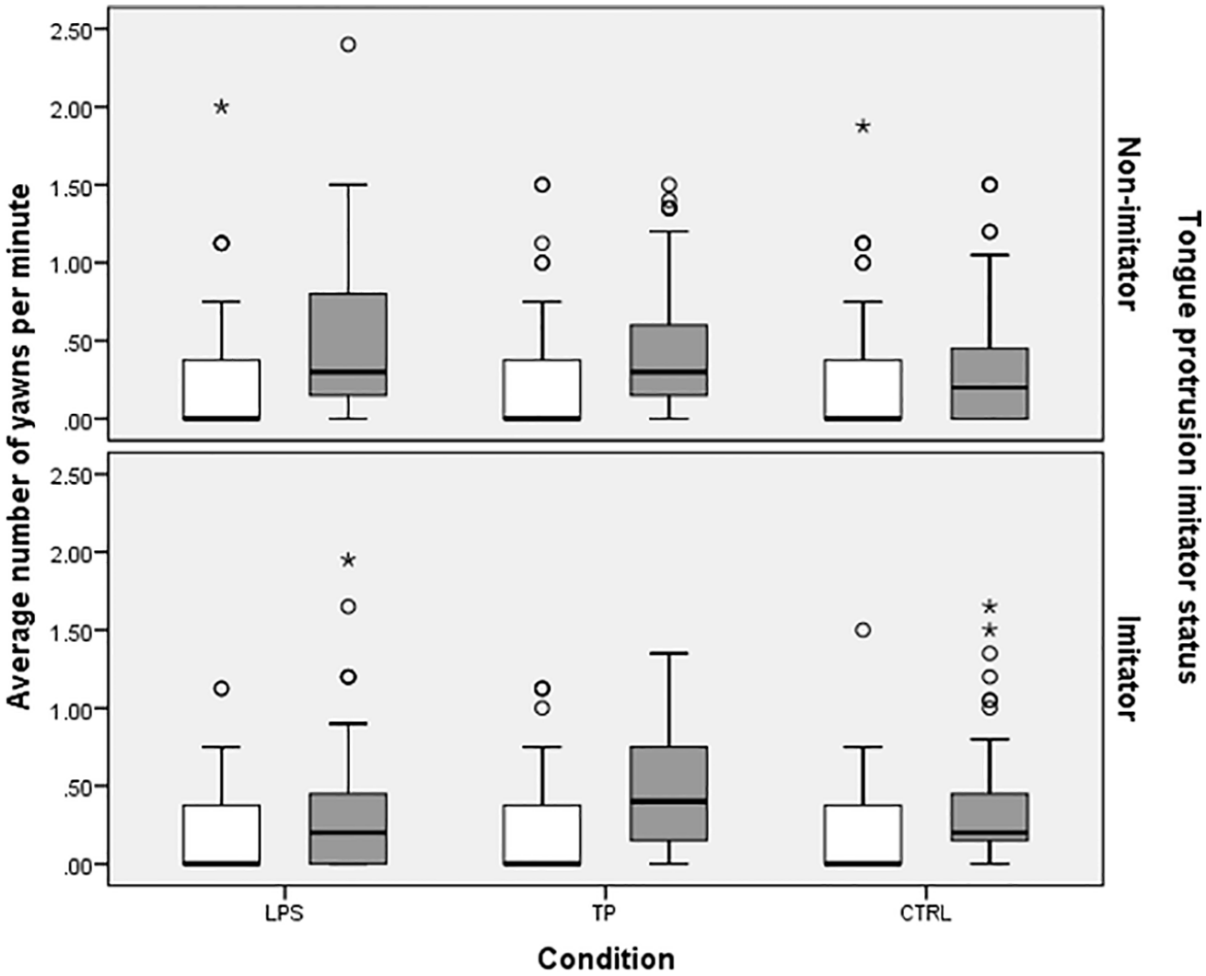
Boxplots of the average rate of yawning per minute during Baseline (white) and Stimulus (grey) for tongue protrusion Non-imitators (top) and Imitators (bottom). LPS = lipsmacking condition, TP = tongue protrusion condition, CTRL = control condition. Boxplots depict 1^st^ and 3^rd^ quartile, line depicts median, whiskers depict 1.5 times inter-quartile range (IQR). Circles represent mild outliers larger than Q3 + 1.5 * IQR, asterisks represent extreme outliers larger than Q3 + 3 * IQR.

Next, we re-ran the same models with a dummy code for tongue protrusion imitator status rather than lipsmacking imitator status. In the first model pooling across conditions, the intercept was significant, b = .15, 95% HDI [.11, .18], pMCMC < .001, indicating that Non-imitators yawned a significant amount. There were no differences between Imitators and Non-imitators, b = -.01, 95% HDI [-.08, .04], pMCMC = .639. Next, we added a dummy code for the face conditions, as well as a condition by imitator status interaction. The intercept of the model was significant, b = .13, 95% HDI [.08, .18], pMCMC < .001, indicating that Non-imitators in the CTRL condition yawned a significant amount. The dummy coded predictor for imitator status was not statistically significant, b = -.02, 95% HDI [-.11, .06], pMCMC = .550, nor was the imitator status by condition interaction, b = .02, 95% HDI [-.07, .10], pMCMC = .727, indicating that Imitators did not yawn more than Non-imitators in either the face conditions or the CTRL condition (Fig 3).

#### Stimulus phase—Lipsmacking Imitators

We ran a Bayesian GLMM predicting yawns during the Stimulus phase with a dummy-coded predictor for lipsmacking imitator status. Non-imitators yawned a significant amount during the Stimulus phase when collapsed across conditions, b = -1.09 (RR = .34), 95% HDI [-1.31, -.86], pMCMC < .001. Imitators did not yawn more than Non-imitators, b = .22 (RR = 1.25), 95% HDI [-.08, .51], pMCMC = .138. Adding dummy-coded predictors for condition (LPS and TP coded separately) to the model, along with condition by imitator status interactions, did not reveal any significant condition by imitator status interactions in yawning during the Stimulus phase, pMCMCs > .501. Thus, Imitators did not yawn more than Non-imitators in the Stimulus phase, and Imitators and Non-imitators yawned similarly across conditions (Fig 2).

#### Stimulus phase—Tongue protrusion Imitators

Next, we re-ran the same models with a dummy code for tongue protrusion imitator status rather than lipsmacking imitator status. Non-imitators yawned a significant amount during the Stimulus phase when collapsed across conditions, b = -1.04 (RR = .35), 95% HDI [-1.27, -.82], pMCMC < .001. Imitators did not yawn more than Non-imitators, b = .04 (RR = 1.04), 95% HDI [-.29, .39], pMCMC = .822. Adding dummy-coded predictors for condition to the model, along with condition by imitator status interactions, did not reveal any significant condition by imitator status interactions in yawning during the Stimulus phase, pMCMCs > .135. Imitators did not yawn more than Non-imitators in the Stimulus phase, and Imitators and Non-imitators yawned similarly across conditions (Fig 3).

#### Change from Baseline phase to Stimulus phase across conditions

We ran Bayesian linear mixed models with dummy codes for condition, imitator status, and their interactions predicting a difference score of yawns per minute during Stimulus versus Baseline (averaged across test day), including random intercepts for subjects. There was a significant increase in the rate of yawns/min for lipsmacking Non-imitators in the CTRL condition, b = .15, 95% HDI [.07, .23], pMCMC < .001. No other terms, including interactions between condition and imitator status, were significant (pMCMCs > .155), indicating that there were no significant differences in the increase in yawn rates between Baseline and Stimulus in any condition or between lipsmacking Imitators and Non-imitators. That is, there was more yawning in the Stimulus phase than in the Baseline phase in all conditions, with no difference in the increase of yawning between Imitators and Non-imitators.

There was also a significant increase in the rate of yawns/min between Baseline and Stimulus for tongue protrusion Non-imitators in the CTRL condition, b = .17, 95% HDI [.10, .26], pMCMC < .001. The dummy code for the LPS condition was also significant, b = .10, 95% HDI [.001, .20], pMCMC = .045, indicating that Non-imitators in the LPS condition increased their yawns/min more than did Non-imitators in the CTRL condition. No other terms, including interactions between condition and imitator status were significant (pMCMCs > .204), indicating that there was no significant effect of imitator status in any condition. In other words, there was no evidence to support the idea that tongue protrusion Imitators increase their yawning rate between Baseline and Stimulus in the TP condition more than tongue protrusion Non-Imitators.

### Associations between yawns and facial gestures

The analyses above reveal that arousal increases in the Stimulus phase of the neonatal imitation test, but there was no indication that arousal was elevated in infants who show a condition-specific increase in facial gestures between the Baseline and Stimulus phase of the matching stimulus condition (i.e., Imitators). Therefore, these data do not support the arousal hypothesis. However, the arousal hypothesis does not only predict a difference in arousal between conditions, it also predicts that arousal causes an increase in facial gestures, which just coincidentally happens more in the experimental (LPS or TP) conditions than the CTRL condition. We therefore analyzed whether changes in yawn rates from Baseline to Stimulus could explain the change in facial gestures (lipsmacking and tongue protrusions) for Imitators or Non-imitators.

#### Lipsmacking gestures

We ran Bayesian linear mixed model with dummy codes for condition, imitator status, and their interactions predicting a difference score of lipsmacking per minute during Stimulus versus Baseline (averaged across test day), including random intercepts for subjects. Lipsmacking Non-imitators in the LPS condition (the intercept of the model) did not have a difference score significantly different from zero, b = -.60, 95% HDI [-1.31, .10], pMCMC = .0944, indicating that their lipsmacking/min did not change between Baseline and Stimulus. However, the difference score for Imitators in the LPS condition was significantly greater than the difference score for Non-imitators in the LPS condition, b = 3.74, 95% HDI [2.71, 4.75], pMCMC < .001, indicating that Imitators’ lipsmacking rate per minute from Baseline to Stimulus increased by 3.74 lipsmacking/min more than Non-imitators’.

Next, we added a difference score of yawns per minute during Stimulus vs Baseline, and its interactions with imitator status and condition, to the model to see if changes in yawn rate from Baseline to Stimulus could explain the change in the lipsmacking rate for Imitators. Neither the effect of yawns (pMCMC = .881) nor the interaction between yawns and imitator status (pMCMC = .610). were significant. The effect of imitator status in the LPS condition remained significant and virtually unchanged, b = 3.58, 95% HDI [2.40, 4.72], pMCMC < .001. Thus, accounting for changes in yawning from Baseline to Stimulus does not explain Imitators’ increase in lipsmacking rates in the LPS condition.

#### Tongue protrusion gestures

We repeated the same two models as above predicting tongue protrusions rather than lipsmacking for tongue protrusion Imitators and Non-imitators while accounting for changes in yawning. Non-imitators in the TP condition significantly decreased their tongue protrusions/min when going from Baseline to Stimulus (the intercept of the model) b = -1.01, 95% HDI [-1.45, -.57], pMCMC < .001. However, the difference score for Imitators in the TP condition was significantly greater than for Non-imitators, b = 2.69, 95% HDI [1.97, 3.41], pMCMC < .001, indicating that Imitators’ tongue protrusions from Baseline to Stimulus increased by 2.69 tongue protrusions/min more than Non-imitators’. To verify that Imitator’s tongue protrusion difference score was significantly greater than zero (since the intercept in the previous model showed a negative effect for Non-imitators), we recoded and re-ran the model with Imitators in the TP condition as the intercept (b = 1.67, 95% HDI [1.08, 2.24], pMCMC < .001). This result indicates that Imitators showed a positive increase in tongue protrusion gestures between Baseline and Stimulus in the TP condition, which was significantly greater than the same difference score for tongue protrusion Non-imitators.

We then added to the model a difference score of yawns per minute during Stimulus vs. Baseline, and its interactions with imitator status and condition, to see if changes in yawn rate from Baseline to Stimulus could explain the change in tongue protrusion rate for Imitators. There was a significant interaction between imitator status and the yawn difference score, indicating that, for Imitators in the TP condition, yawn difference scores were positively associated with tongue protrusion difference scores (b = 2.13, 95% HDI [.32, 3.97], pMCMC = .022. That is, increases in tongue protrusion rate from Baseline to Stimulus were associated with increases in yawn rate for Imitators. However, controlling for yawns *did not* eliminate the effect of imitator status in the model (b = 2.22, 95% HDI [1.39, 3.06], pMCMC < .001). In other words, even after controlling for yawning, TP imitators still showed a larger increase in tongue protrusions in the Stimulus phase of the TP condition than Non-imitators; the changes in yawning from Baseline to Stimulus do not fully explain Imitators’ increase in tongue protrusion rates in the TP condition.

## Discussion

The results above suggest that arousal may play a role during neonatal imitation assessments. In particular, while we found no difference in arousal among conditions during the Baseline phase, infants were significantly more likely to yawn during the Stimulus phase when a dynamic stimulus was presented. This result is inline with reports by Nagy and Molnar [18], who found that human infants’ heart rates accelerated when they were viewing and imitating a human tongue protrusion model compared to a baseline measurement. Moreover, our data indicate that infants were more aroused when seeing dynamic facial stimuli compared to the dynamic non-social control stimulus. These findings suggest that the experimental facial conditions of our neonatal imitation task indeed increased infant’s levels of arousal. According to the arousal hypothesis, this increase of arousal could lead to an increase in facial gestures. However, infants who displayed an increase in facial gestures during the experimental conditions (Imitators) did not show a corresponding increase in arousal in the experimental conditions when compared to either the control condition, or to infants who did not show an increase in facial gestures (Non-imitators). Finally, both LPS and TP Imitators showed a significant increase in facial gestures between the Baseline and Stimulus phases of their matching facial gesture conditions even after controlling for arousal; together these findings suggests that Imitators’ propensity to match facial gestures is based on abilities that go beyond mere arousal. This study adds to a growing body of recent evidence in human and nonhuman primates that suggest newborns’ matching abilities are inconsistent with an arousal hypothesis [19, 23, 35, 37, 47].

The current study and its conclusion could be strengthened in several ways. For example, a confirmatory measure of arousal, such as heart rate or galvanic skin response, would further support the idea that yawning is a good proxy of arousal. Moreover, parallel findings in another population, e.g. human neonates, would speak to the generalizability of the current findings and point to a common mechanism of neonatal imitation, perhaps across evolutionary lines. Finally, despite high inter-observer agreement, a more objective measure of facial movements, including fine-grained analyses of facial muscle activation as used by the Facial Action Coding system, FACS [48–49] for humans and the MaqFACS [50] for macaques, could rule out any potential bias of subjective observers. These aspects should be considered in future studies.

Even though we argue that arousal cannot account for neonatal imitation performances on its own, the current data nonetheless indicate that infant arousal is a factor during these assessments. For example, infants appeared to become more aroused with increasing age and/or test sessions, even over the course of just one week, the causes of which are currently unknown. Careful experimental manipulation of these variables could shed further light on this issue. One possibility is that as infants’ visual acuity improves [51], they are better able to perceive and process their surroundings, which could lead to more engagement with and responsiveness to visual stimuli. Neonatal imitation assessments are currently conducted with human infants within minutes after birth (e.g. [4]) up to 12 weeks old or even older (e.g. [52]); infant age, and infants’ potential for arousal, should therefore be taken into consideration during neonatal assessments. Secondly, the fact that dynamic stimuli appear more arousing than static stimuli, and in particular that dynamic social stimuli seem more arousing than a dynamic non-social stimulus, should be addressed when designing and analyzing neonatal imitation data. For example, if a study were to test infants merely in a social Baseline and Stimulus phase to assess imitation, then investigators might be more likely to classify infants as Imitators since infants are expected to show higher arousal levels (and thus more facial gestures) during the Stimulus phase. Equally, if a study only included dynamic Stimulus phases of social and non-social stimuli, then infants are also more likely to be classified as Imitators since dynamic social stimuli appear to be more arousing than dynamic non-social stimuli. Conversely, it is not enough to show that a certain type of stimulus (e.g. music or blinking lights) increases infants’ facial gesturing to conclude that neonatal imitation is merely an artifact of arousal; one must also test infants in the actual imitation condition to demonstrate that in the imitation condition, infants do not produce facial gestures *above* general arousal levels. Thus, both a static Baseline as well as a non-social control condition should be included, whenever possible, in neonatal imitation assessments, and infants’ *increase* in gesture rate between Baseline and Stimulus as well as between experimental and control condition should be taken into account. These testing conditions represent the most sensitive assessment of imitation-specific action matching and may be particularly useful for studies examining individual differences in imitative skills [11].

## Supporting information

S1 Video Illustration of lipsmacking condition. This video illustrates a 6-day-old infant macaque lipsmacking and yawning during the Stimulus phase of the Lipsmacking condition. The model (not shown) is to the left of the screen.
(MOV)

S2 Video Illustration of control condition. This video illustrates a 7-day-old infant macaque lipsmacking and yawning during the Stimulus phase of the Control condition. The disk (not shown) is to the left of the screen.
(MOV)

S1 Fig Illustration of the phases of a neonatal imitation test (Lipsmacking condition). (TIF)

S1 Table Raw data file. (XLSX)

## References

[1] Cowart BJ. Development of taste perception in humans: Sensitivity and preference throughout the lifespan. Psychol. Bull. 1981; 90: 43–73. 7267897

[2] Mizuno K, Mizuno N, Shinohara T, Noda M. Mother-infant skin-to-skin contact after delivery results in early recognition of own mother's milk odour. Acta Paediatr. 2004; 93: 1640–5. 15841774 10.1080/08035250410023115

[3] Meltzoff AN, Moore MK. Imitation of facial and manual gestures by human neonates. Science. 1977; 198: 75–78. doi: 10.1126/science.198.4312.75 17741897 10.1126/science.198.4312.75

[4] Meltzoff AN, Moore MK. Newborn infants imitate adult facial gestures. Child Dev. 1983; 54: 702–709. 6851717

[5] Koepke JE, Hamm M, Legerstee M, Russell M. Neonatal imitation: two failures to replicate. Infant Behav Dev. 1983; 6: 97–102.

[6] Field TM, Woodson R, Cohen D, Greenberg R, Garcia R, Collins K. Discrimination and imitation of facial expressions by term and preterm neonates. Infant Behav Dev. 1983; 6: 485–489.

[7] Meltzoff AN, Moore MK. Imitation in newborn infants: exploring the range of gestures imitated and the underlying mechanisms. Dev Psychol. 1989; 25: 954–962. doi: 10.1037/0012-1649.25.6.954 25147405 10.1037/0012-1649.25.6.954 PMC4137867

[8] Nagy E, Kompagne H, Orvos H, Pal A, Molnar P, Janszky I, et al Index finger movement imitation by human neonates: motivation, learning, and left hand preference. Pediatr Res. 2005; 58: 749–753. doi: 10.1203/01.PDR.0000180570.28111.D9 16189204 10.1203/01.PDR.0000180570.28111.D9

[9] Nagy E, Pal A, Orvos H. Learning to imitate individual finger movements by the human neonate. Dev Sci. 2014; 17: 841–857. doi: 10.1111/desc.12163 24754667 10.1111/desc.12163

[10] Nagy E. The newborn infant: a missing stage in developmental psychology. Infant Child Dev. 2011; 20: 3–19.

[11] Simpson EA, Murray L, Paukner A, Ferrari PF. The mirror neuron system as revealed through neonatal imitation: presence from birth, predictive power and evidence of plasticity. Phil Trans R Soc B, 2014; 369: 20130289 doi: 10.1098/rstb.2013.0289 24778381 10.1098/rstb.2013.0289 PMC4006187

[12] Anisfeld M. Neonatal imitation. Dev Rev. 1991; 11: 60–97.

[13] Anisfeld M. Only tongue protrusion modeling is matched by neonates. Dev Rev. 1996; 16: 149–161.

[14] Galef BG Jr. Imitation in animals: History, definition, and interpretation of data from the psychological laboratory In: Social learning: Psychological and biological perspectives, ed. Zentall TR & Galef BG Jr. Erlbaum. 1988; p 3–28.

[15] Meltzoff AN, Moore MK. Explaining facial imitation: a theoretical model. Early Dev Parent, 1997; 6: 179–192. doi: 10.1002/(SICI)1099-0917(199709/12)6:3/4<179::AID-EDP157>3.0.CO;2-R 24634574 10.1002/(SICI)1099-0917(199709/12)6:3/4<179::AID-EDP157>3.0.CO;2-R PMC3953219

[16] Jones SS. Imitation or exploration? Young infants matching of adults’ oral gestures. Child Dev. 1996; 67: 1952–1969. 9022224

[17] Jones SS. Exploration of imitation? The effect of music on 4-week-old infants’ tongue protrusions. Infant Behav Dev.2006; 29: 126–130. doi: 10.1016/j.infbeh.2005.08.004 17138267 10.1016/j.infbeh.2005.08.004

[18] Nagy E, Molnar P. Homo imitans or homo provocans? Human imprinting model of neonatal imitation. Infant Behav Dev. 2004; 27: 54–63.

[19] Nagy E, Pilling K, Orvos H, Molnar P. Imitation of tongue protrusion in human neonates: specificity of the response in a large sample. Dev Psychol, 2013; 49: 1628–1638. doi: 10.1037/a0031127 23231691 10.1037/a0031127

[20] Ferrari PF, Visalberghi E, Paukner A, Fogassi L, Ruggiero A, Suomi SJ. Neonatal imitation in rhesus macaques. PLoS Biol. 2006; 4: e302 doi: 10.1371/journal.pbio.0040302 16953662 10.1371/journal.pbio.0040302 PMC1560174

[21] Bard KA. Neonatal imitation in chimpanzees (Pan troglodytes) tested with two paradigms. Animal Cogn. 2007; 10: 233–242. 10.1007/s10071-006-0062-3 17180698

[22] Paukner A, Ferrari PF, Suomi SJ. Delayed imitation of lipsmacking gestures by infant rhesus macaques. PLoS ONE, 2011; 6: e28848 doi: 10.1371/journal.pone.0028848 22174913 10.1371/journal.pone.0028848 PMC3236225

[23] Simpson EA, Paukner A, Suomi SJ, Ferrari PF. Visual attention during neonatal imitation in newborn macaque monkeys. Dev Psychobiol. 2014; 56: 864–870. doi: 10.1002/dev.21146 23794178 10.1002/dev.21146 PMC3805767

[24] Maestripieri D, Schino G, Aureli F, Troisi A. A modest proposal: Displacement activities as an indicator of emotions in primates. Anim. Behav, 1992; 44: 967–979.

[25] Argiolas A, Melis MR. The neuropharmacology of yawning. Eur J Pharmacol. 1998: 343: 1–16. 9551709 10.1016/s0014-2999(97)01538-0

[26] Deputte BL. Ethological study of yawning in primates. 1. Quantitative analysis and study of causation in two species of old world monkeys (Cercocebus albigena and Macaca fascicularis). Ethology. 1994; 98: 221–245.

[27] Hadidian J. Yawning in an old world monkey (Macaca nigra). Behav, 1980: 75: 133–147.

[28] Bertrand M. The behavioural repertoire of the stumptail macaque. Karger: Basel 1996.

[29] Maestripieri D, Schino G, Aureli F, Troisi A. A modest proposal: displacement activities as an indicator of emotions in primates. Animal Behav. 1992; 44: 967–979.

[30] Baenninger R, Binkley S, Baenninger M. Field observations of yawning and activity in humans. Physiol Behav. 1996; 59: 421–425. 8700941 10.1016/0031-9384(95)02014-4

[31] Leone A, Ferrari PF, Palagi E. Different yawns, different functions? Testing social hypotheses on spontaneous yawning in Theropithecus gelada. Sci Rep. 2014; 4: 4010 doi: 10.1038/srep04010 24500137 10.1038/srep04010 PMC5379258

[32] Liang AC, Grace JK, Tompkins EM, Anderson DJ. Yawning, acute stressors, and arousal reduction in Nazca booby adults and nestlings. Physio Behav. 2015; 140: 38–43. 10.1016/j.physbeh.2014.11.029 25498600

[33] Giganti F, Hayes MJ, Akilesh MR, Salzarulo P. Yawning and behavioral states in premature infants. Dev Psychobiol, 2002; 41: 289–296. doi: 10.1002/dev.10047 12325143 10.1002/dev.10047

[34] Holsti L, Grunau RE, Obserlander TF, Whitfield MF, Weinberg J. Body movements: an important additional factor in discriminating pain from stress in preterm infants. Clin J Pain, 2005; 21: 491–498. 16215334 10.1097/01.ajp.0000146163.30776.44 PMC1852478

[35] Simpson EA, Miller G, Ferrari PF, Suomi SJ, Paukner A. Neonatal imitation and early social experience predict gaze following abilities in infant macaques. Sci Rep, 2016; 6: 20233 doi: 10.1038/srep20233 26831763 10.1038/srep20233 PMC4735292

[36] Ferrari PF, Paukner A, Ruggiero A, Darcey L, Unbehagen S, Suomi SJ. Interindividual differences in neonatal imitation and the development of action chains in rhesus macaques. Child Dev. 2009; 80: 1057–1068. doi: 10.1111/j.1467-8624.2009.01316.x 19630893 10.1111/j.1467-8624.2009.01316.x PMC3648862

[37] Kaburu SSK, Paukner A, Simpson EA, Suomi SJ, Ferrari PF. Neonatal imitation predicts infant rhesus macaque (Macaca mulatta) social and anxiety-related behaviours at one year. Sci. Rep. 2016; 6: 34997 doi: 10.1038/srep34997 27725768 10.1038/srep34997 PMC5057109

[38] Simpson EA, Sclafani V, Paukner A, Hamel AF, Novak MA, Meyer JS, et al Inhaled oxytocin increases positive social behaviors in newborn macaques. Proc Nat Acad Sci. 2014; 111: 6922–6927. doi: 10.1073/pnas.1402471111 24778211 10.1073/pnas.1402471111 PMC4024859

[39] Simpson EA, Nicolini Y, Shetler M, Suomi SJ, Ferrari PF, Paukner A. Experience-independent sex differences in newborn macaques: Females are more social than males. Sci Rep, 2016; 6: 19669 doi: 10.1038/srep19669 26794858 10.1038/srep19669 PMC4726418

[40] Paukner A, Simpson EA, Ferrari PF, Mrozek T, Suomi SJ. Neonatal imitation predicts how infants engage with faces. Dev Sci. 2014; 17: 833–840. doi: 10.1111/desc.12207 24995706 10.1111/desc.12207 PMC4211944

[41] Ferrari PF, Vanderwert RE, Paukner A, Bower S, Suomi SJ, Fox NA. Distinct EEG amplitude suppression to facial gestures as evidence for a mirror mechanism in newborn monkeys. J Cognitive Neurosci. 2012; 24: 1165–1172. 10.1162/jocn_a_00198 PMC3639488 22288390

[42] Bolker BM, Brooks ME, Clark CJ, Geange SW, Poulsen JR, Stevens MHH, et al Generalized linear mixed models: a practical guide for ecology and evolution. Trends Ecol Evol. 2009; 24: 127–135. doi: 10.1016/j.tree.2008.10.008 19185386 10.1016/j.tree.2008.10.008

[43] Atkins DC, Baldwin SA, Zheng C, Gallop RJ, Neighbors C. A tutorial on count regression and zero-altered count models for longitudinal substance use data. Psychol Addict Behav. 2013; 27: 166 doi: 10.1037/a0029508 22905895 10.1037/a0029508 PMC3513584

[44] R Development Core Team. (2014). R: A language and environment for statistical computing: R Foundation for Statistical Computing, Vienna, Austria URL: http://www.R-project.org/.

[45] Hadfield JD. MCMC methods for multi-response generalized linear mixed models: the MCMCglmm R package. J Stat Softw. 2010; 33: 1–22.20808728

[46] Goy RW Resko JA. Gonadal hormones and behavior of normal and pseudohermaphroditic nonhuman female primates. Recent Prog Horm Res. 1972; 28: 707–733. 4631621

[47] Meltzoff AN, Murray L, Simpson EA, Heimann M, Nagy E, Nadel J, et al Re-examination of Oostenbroek et al. (2016): Evidence for neonatal imitation of tongue protrusion. Dev. Sci. 10.1111/desc.12609 PMC6710010 28952202

[48] Ekman P, Friesen WV. Measuring facial movement. Environ Psychol Nonverbal Behav. 1976; 1: 56–75.

[49] Messinger DS, Mahoor MH, Chow SM, Cohn JF. Automated measurement of facial expression in infant-mother interaction: A pilot study. Infancy. 2009: 14: 285–305 doi: 10.1080/15250000902839963 19885384 10.1080/15250000902839963 PMC2746084

[50] Parr LA, Waller BM, Burrows AM, Gothard KM, Vick SJ. MaqFACS: A muscle-based facial movement coding system for the rhesus macaque. Am J Phys Anthropol, 2010; 143: 625–630. doi: 10.1002/ajpa.21401 20872742 10.1002/ajpa.21401 PMC2988871

[51] Ordy JM, Latanick A, Samorajski T, Massopust LC. Visual acuity in newborn primate infants. Exp Biol Med. 1964; 115: 677–680. 10.3181/00379727-115-29004 14155796

[52] Jacobson SW. Matching behavior in the young infant. Child Dev. 1979; 50: 425–430. 487882

