# Supplementary figures and images for: Testing the arousal hypothesis of neonatal imitation in infant rhesus macaques
Source: PLoS One. 2017 Jun 15;12(6):e0178864. doi: 10.1371/journal.pone.0178864 (PMC5472263; doi:10.1371/journal.pone.0178864)

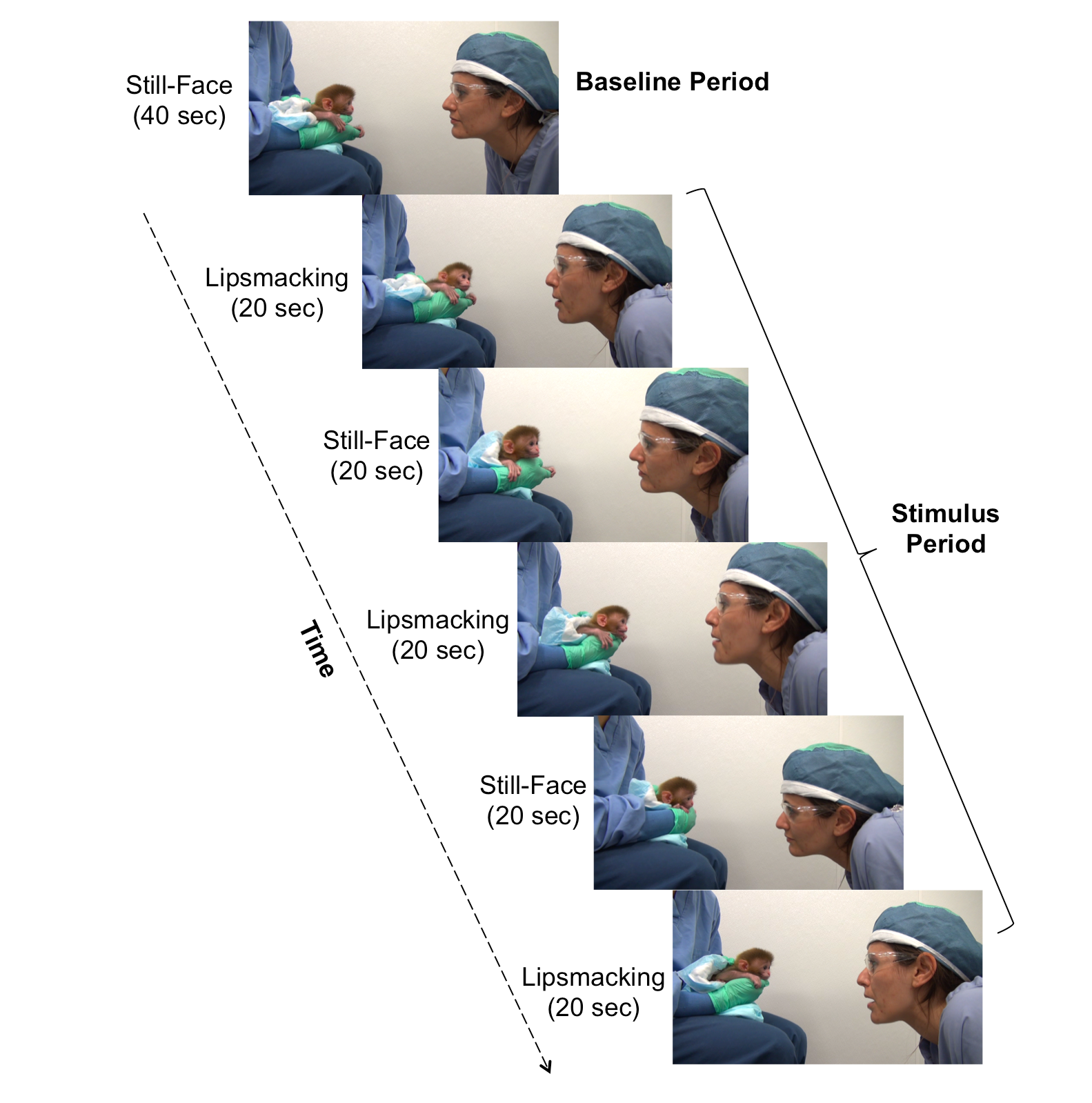

Supplement: S1 Fig — (TIF) [file pone.0178864.s003.tif]
